# The Approach to a Child with Dysmorphic Features: What the Pediatrician Should Know

**DOI:** 10.3390/children11050578

**Published:** 2024-05-10

**Authors:** Silvia Ciancia, Simona Filomena Madeo, Olga Calabrese, Lorenzo Iughetti

**Affiliations:** 1 Pediatric Unit, Department of Medical and Surgical Sciences for Mothers, Children and Adults, University of Modena and Reggio Emilia, Largo del Pozzo 71, 41124 Modena, Italy; 2 Medical Genetics Unit, Department of Medical and Surgical Sciences for Mothers, Children and Adults, University of Modena and Reggio Emilia, 41124 Modena, Italy

**Keywords:** genetic syndrome, congenital malformation, dysmorphic feature, intellectual disability, neurodevelopmental delay, genetic testing

## Abstract

The advancement of genetic knowledge and the discovery of an increasing number of genetic disorders has made the role of the geneticist progressively more complex and fundamental. However, most genetic disorders present during childhood; thus, their early recognition is a challenge for the pediatrician, who will be also involved in the follow-up of these children, often establishing a close relationship with them and their families and becoming a referral figure. In this review, we aim to provide the pediatrician with a general knowledge of the approach to treating a child with a genetic syndrome associated with dysmorphic features. We will discuss the red flags, the most common manifestations, the analytic collection of the family and personal medical history, and the signs that should alert the pediatrician during the physical examination. We will offer an overview of the physical malformations most commonly associated with genetic defects and the way to describe dysmorphic facial features. We will provide hints about some tools that can support the pediatrician in clinical practice and that also represent a useful educational resource, either online or through apps downloaded on a smartphone. Eventually, we will offer an overview of genetic testing, the ethical considerations, the consequences of incidental findings, and the main indications and limitations of the principal technologies.

## 1. Introduction

The estimation of the incidence of genetic disorders dates back to 1988, when Baird et al., examining the database of the population-based British Columbia Health Surveillance Registry, calculated that 5–8% of the population was affected by a genetic disease [1]. Several of these genetic disorders are associated with physical malformations and dysmorphic features suggestive of a specific diagnosis or of a spectrum. Feeding problems, hypotonia, cardiovascular defects, ocular manifestations, skeletal malformations, and kidney anomalies can also suggest a genetic condition [2,3,4,5]. Through the recognition of red flags, a prompt diagnostic work-up can be set up, and a timely diagnosis can be achieved.

While some genetic syndromes are immediately recognizable and relatively common, and pediatricians are familiar with them (for example achondroplasia) [6], others can be very rare; the phenotype can be mild or not unique, and the diagnosis is challenging. In particular, this can be due to different phenotypic expressions of the same genotype [7,8]. Moreover, thanks to the development of technologies for extensive genome sequencing, a wide spectrum of genetic disorders is known nowadays, making the chapter of genetic disorders broader and broader. The pediatrician is not asked to recognize and diagnose all possible genetic syndromes. However, he/she must identify patients requiring specific investigations and start the diagnostic work-up to guarantee that the patient will receive a timely diagnosis and a prompt screening for comorbidities. For example, in Noonan syndrome, cardiovascular involvement is present in a high percentage of individuals, mainly as congenital heart disease or hypertrophic cardiomyopathy [9]. An early diagnosis allows for the screening of cardiovascular involvement and the start of the appropriate treatment and follow-up. Noonan syndrome and some other genetic syndromes are also associated with an increased risk of cancer in childhood. Examples are Costello syndrome and RASopathies, Sotos, Rubinstein–Taybi, Gorlin–Goltz, and Bloom syndrome [10,11,12], all of which require careful monitoring to prevent the development of advanced malignancies. In this review, we will highlight the main information that should be acquired about the family and medical history, we will give some input about the physical examination of a child presenting with dysmorphic features. We will also share information about some tools that can be a good support for the clinician in the diagnosis of genetic disorders and their comprehension. Furthermore, we will discuss the principal genetic technologies used to achieve genetic diagnoses.

## 2. Clinical Work-Up

### 2.1. Family and Medical History

A detailed family history should always be collected, asking for information on at least the last three generations. In case of a positive family history, a family tree reconstruction is needed. It is not only important to investigate consanguinity, people affected by diagnosed genetic disorders, and congenital anomalies (e.g., skeletal malformations) but also intellectual disability, neurodevelopmental delay, early-onset obesity and/or hyperphagia, visual deficits, and early hearing loss. Moreover, it is important to collect information about early unexplained deaths, repeated spontaneous miscarriages, fetal deaths, or stillbirths. The obstetric history should be analyzed, looking for maternal diseases, the use of medications, the abuse of alcohol or drugs, smoking, and infections acquired during pregnancy (e.g., TORCH complex: toxoplasmosis, rubella, cytomegalovirus, herpes virus, and also cytomegalovirus, Zika virus, parvovirus B19, lymphocytic choriomeningitis virus, treponema pallidum, and many others); intrauterine growth, abnormalities of amniotic fluid quantity, non-invasive prenatal screening (NIPS) results, ultrasound abnormalities, and the presence of fetal movements should be questioned. Children affected by genetic conditions can present with several problems as newborns, and adaptation to extrauterine life can be difficult; length, weight, head circumference, and percentiles at birth should be acquired. Irregular acquisition of developmental milestones and abnormal growth also represent red flags. Also, internal organ dysfunction and congenital anomalies (for example congenital heart defects, congenital diaphragmatic hernia, kidney malformations, hypospadias) can be associated with genetic syndromes.

### 2.2. Physical Examination

After the collection of the family and medical history, the physical examination represents the next fundamental step to look for hints that can support the clinician in the diagnostic process. When there is suspicion of a genetic condition, the child should be examined thoroughly. The pediatrician is not asked to be able to perform a detailed dysmorphological examination; however, basic knowledge in this field can help in the search of red flags. Short (and disproportioned) stature, impaired growth, and overgrowth can be easily assessed through the following measurements: height (or length for children aged less than 2 years), sitting height, arm span (the distance from the middle fingertip of the right hand to the middle fingertip of the left hand), sitting height/height ratio, cranial circumference (macrocephaly is defined if cranial circumference is above the 97° percentile, microcephaly if cranial circumference is below the 3° percentile), waist and hip circumference (in case of obese children), weight, and body mass index (BMI, calculated as the ratio of the weight, expressed in kilograms, and the square height, expressed in meters) [13,14,15,16,17,18,19,20,21,22] (https://www.who.int/tools/child-growth-standards/standards, accessed on 5 May 2024), (https://www.cdc.gov/growthcharts/clinical_charts.htm, accessed on 5 May 2024).

Afterward, the different body regions should be analyzed. For example, limb malformation (e.g., forearm defects, genu varum or valgus), hand malformations (e.g., cleft hand, clinodactyly, camptodactyly, syndactyly, polydactyly, broad thumbs, hypoplastic thumbs), and foot malformations (e.g., cleft foot, clubfoot, polydactyly, syndactyly, broad first toes) are often associated with a genetic syndrome. Also, alterations in the shape of the thorax, or even malformations of genitalia, can be informative. If several abnormalities are present at the same time, characteristic associations can be recognized as vertebral defects, anal atresia, cardiac defects, trachea-esophageal fistula, renal anomalies, and limb anomalies (VACTERL), which clearly indicates an underlying condition. Of note, radiological examinations, such as radiography of the spine, thorax, or limbs, or even total-body radiography, can help in better identifying skeletal malformations.

However, a distinctive tract of several genetic syndromes is often represented by a characteristic face. At first look, everybody is able to recognize a face with an abnormal appearance. The measurements of segments and diameters and their comparison with normative data are left to the geneticist; however, the pediatrician can make a qualitative description. For example, a broad face is characterized by an apparent increase in width, a long face by an apparent increase in length, and a coarse face by heavy features and thickened skin, whether associated with thickened bones or not. Also, a face can be perceived as triangular, square, small, etc.

**Figure 1:**
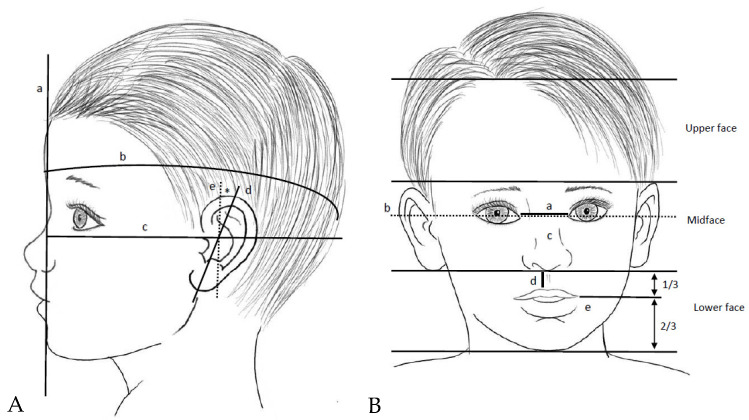
Facial regions (**A**): (a) sagittal line passing through the glabella; (b) head circumference; (c) Frankfurt plane passing under the inferior margin of the orbit and the ear canal; (d) longitudinal axis of the ear; (e) perpendicular to the Frankfurt plane; * in normally set ears this angle is around 20°. (**B**): (a) intercanthal distance; (b) line passing through the inner canthi and extended to the ears; (c) nasal bridge; (d) philtrum; (e) oral commissure.

As shown in Figure 1, the face can be divided into three regions (upper, mid, and lower face). In the upper face, the main things to dwell on are the hair and the hairline (high, low, hair balding) and the forehead (broad, narrow, prominent, sloping, with a depressed/prominent glabella), with some features being more evident in the profile view (for example the frontal bossing). In the midface, the position and shape of the eyes, eyebrows, nose, and ears should be noted. Some imaginary reference lines can help; for example, the distance between the inner canthi of the eyes (usually equal to the width of each eye) can be shorter or greater (respectively hypotelorism and hypertelorism), and ears can be low-set (the upper part of the ears is below a line passing through the inner canthi and extended in the direction of the ears) or abnormally rotate (Figure 1A,B). If the upper lid margin covers part of the pupil, the patient has ptosis, while a fold of skin starting above the upper eyelid and arching downward to cover the inner canthus is called epicanthus. The nasal bridge can be narrow, wide, depressed, or prominent; the columella can have a high/low insertion and can be broad or short; the nares can be anteverted, enlarged, narrow, or single; the nasal tip can be bulbous, depressed, deviated, or narrow; and the philtrum can be long or short. In the lower face, the mouth and the chin are the main parts to observe. The mouth can be wide, narrow, downturned, or upturned and the vermilions can be thin, thick, or everted. Also, the oral cavity should be explored, looking at mucosal abnormalities, tongue, cleft, and teeth (for example teeth abnormalities are commonly present in the most severe forms of osteogenesis imperfecta). The chin can be short, long, or pointed, and the mandibula can be hypoplastic (micrognathia) or can protrude forward (prognathism).

For example, Silver–Russell syndrome patients present with a triangular-shaped face, relative macrocephaly, a protruding forehead, low-set and/or posteriorly rotated ears, micrognathia, a down-turned mouth, delayed dental eruption and microdontia (small teeth), body asymmetry, short stature, and a low BMI [23]. Noonan syndrome patients show a large head, a tall and prominent forehead, arched and diamond-shaped eyebrows, hypertelorism, ptosis, low-set and posteriorly rotated ears, and a bulbous upturned nose tip [24]. Prader–Willi syndrome patients present with a narrow forehead, almond-shaped eyes, a down-turned mouth, straight borders of the inner legs, and obesity [25]. Beckwith–Wiedemann syndrome patients present with macroglossia, overgrowth, hemihyperplasia, and abdominal wall defects [26]. Kabuki syndrome patients show long palpebral fissures and eversion of the lateral third of the lower eyelid, large and arched eyebrows, large and prominent ears, a short columella and depressed nasal tip, and skeletal anomalies of the hands [27].

It is important to remember that facial features can be subtle, and they can change over time, being more evident in early childhood for some syndromes or more evident in adulthood for others. Also, a few dysmorphic traits should not raise the suspicion of a genetic syndrome if they are isolated, as some variants of normal morphology are non-pathological.

In the next chapter, we will list some tools that can be supportive for clinical practice or used as educational material.

## 3. Supportive Technological Tools

### 3.1. Human Malformation Terminology (National Human Genome Research Institute)

Clinicians and, in particular, dysmorphologists, use several terms to describe body parts and their morphologies. The human malformation terminology website has been created to standardize these terms and their definitions, helping clinicians to communicate and exchange information by speaking a “common language”. The website contains six windows analyzing the terminology relative to head and face; periorbital region; ears, nose, and philtrum; lips, mouth, and oral region; and hands and feet. The website is enriched with definitions and pictures or drawings of each dysmorphic feature, and it is open access (https://elementsofmorphology.nih.gov/, accessed on 5 May 2024).

### 3.2. ACT Sheets and Algorithms

The ACT sheets and algorithms are free-to-access resources offered by the American College of Medical Genetics and Genomics (ACMG) with a primarily educational intent and conceived to support clinicians in the decision-making process. There are seven sections, including newborn screening, non-invasive prenatal screening (NIPS), carrier of genetic mutations, diagnostic tests, positive family history, secondary findings, and transition to adult care. With the exception of newborn screening, the sections include only a few of the most common disorders; however, the sheets are written in simple language and offer brief and clear information. Each ACT sheet also include links to resources for additional information (https://www.acmg.net/ACMG/Medical-Genetics-Practice-Resources/ACT_Sheets_and_Algorithms.aspx, accessed on 5 May 2024).

### 3.3. Online Mendelian Inheritance in Man

Online Mendelian Inheritance in Man (OMIM) is an open-access online database of human genes and genetic disorders that is updated daily. It contains information on all Mendelian disorders and over 16,000 genes. The database was initiated in the early 1960s by Dr. Victor McKusick, and it has been enriched over the decades after the discovery of new genes and disorders, under the direction of the staff of the Johns Hopkins University, Baltimore, U.S.A. [28,29]. The search box can be interrogated with clinical features, phenotypes, and genes. Through the clinical synopsis function, it is possible to search for disorders affecting one or more systems, growth, endocrine or metabolic features, or laboratory abnormalities or those characterized by prenatal manifestations. For each disorder, the phenotype–gene relation and a detailed description are provided. For every chromosome, a gene map is given, and the search for a specific locus can be made. For each gene, the list of allelic variants is provided as a table or as a link to ClinVar, which is a free-to-access archive on the relation between human genetic variants and phenotypes [30] (https://omim.org/, accessed on 5 May 2024), (https://www.ncbi.nlm.nih.gov/clinvar/, accessed on 5 May 2024).

### 3.4. Risk Calculators

The Cleveland Clinic offers an online risk calculator to estimate the risk a person has of carrying a PTEN mutation (tumor suppressor gene, chromosome 10q23.3). Mutations of this gene are inherited as autosomal dominant with high penetrance and have been increasingly recognized in several syndromes, e.g., Cowden syndrome (adulthood) and Bannayan–Riley–Ruvalcaba syndrome (childhood). Both syndromes are associated with multisystem involvement, macrocephaly, overgrowth, hamartomas, cancers, and dermatological, neurological, and gastrointestinal manifestations. The Cleveland Clinic offers both adult and pediatric scores to assess the pre-genetic testing risk of individuals [31,32], (https://www.lerner.ccf.org/gmi/ccscore/, accessed on 5 May 2024).

The Marfan Foundation offers a systemic score calculator to assess the risk a person has of being affected by Marfan syndrome. In 2010, the Ghent nosology for Marfan syndrome was revised, and aortic root aneurysm and ectopia lentis (dislocated lenses) were identified as cardinal features. Family history must be considered; however, if family history is negative, these two features are sufficient to make the diagnosis [33]. The score calculator includes sixteen features and their relative scores. A score ≥ 7 is positive (https://www.marfan.org/dx/score, accessed on 5 May 2024).

### 3.5. Face2Gene

In recent years, advances in artificial intelligence have led to the development of tools like Face2Gene (FDNA, Inc., Boston, MA, USA). Face2Gene represents an aid for clinicians to recognize most common genetic syndromes based on facial gestalt (information contained in the facial morphology derived from the analysis of different parts such as eyes, eyebrows, ears, nose, mouth, forehead, jawline, and chin). The Face2Gene app can be downloaded on a smartphone or used online, access is protected by a secret password, and only recognized healthcare providers can subscribe (free of charge). It is based on computer vision and deep-learning algorithm technology that quantifies similarities between the patient and thousands of known syndromes. After a picture of the patient is taken, facial landmarks are detected and used to crop the face into multiple regions that are compared with the pictures contained in the database (the Winter–Baraitser Dysmorphology Database, previously known as London Medical Database) to score the similarity [34]. A list of 30 syndromes is suggested, from the most to the least probable, and a bar plot shows the level of gestalt similarity (from low to high). Also, clinical features can be added to refine the phenotype. Information about each suggested syndrome is provided. The software automatically extracts de-identified data from individual patient facial photos that cannot be reverse engineered into an identifiable facial photo to assure patients’ privacy. Original photos are encrypted and stored securely in a separate area of the database that is only available to the individual clinician or researcher who submitted the case. The app has already been validated in some studies that have also included non-Caucasian patients to take into account face variations that occur in different ethnic groups with good results [35,36,37,38,39,40,41], (https://www.face2gene.com/, accessed on 5 May 2024).

### 3.6. DECIPHER

The DECIPHER database allows data sharing from almost 40,000 patients affected by chromosome abnormalities and the comparison of their phenotypic and genotypic data. More than 270 international academic clinical centers contribute to the database. Through the phenotype browser, it is possible to search for genetic diagnosis starting from a specific phenotype. DECIPHER also offers strategies to promote collaboration among clinicians and researchers. It is free of charge [42,43] (https://www.deciphergenomics.org/, accessed on 5 May 2024).

### 3.7. POSSUMweb

Pictures Of Standard Syndromes and Undiagnosed Malformations (POSSUM) is a dysmorphology database that includes more than 30,000 images (photos, X-rays, scans, diagrams, and histological samples) referring to more than 5000 syndromes. It is offered by the Victorian Clinical Genetics Service and the Murdoch Children’s Research Institute in Melbourne (Australia). It is designed as a diagnostic tool, to support clinicians in the recognition of genetic syndromes; however, it is also used for teaching and education. The main limitation is that after a free trial of 21 days, a subscription is needed (https://www.possum.net.au/, accessed on 5 May 2024).

## 4. Genetic Testing

The achievement of a genetic diagnosis can provide information about the prognosis and the risk of comorbidities and give realistic expectations about the quality of life and life expectancy of the child. Also, it can help some parents overcome the feeling of guilt that they often harbor and allow them to seek peer groups to share their experiences. Moreover, it can guide prenatal counseling.

The choice of genetic testing is beyond the role of a general pediatrician, and it is ordered by a geneticist or a pediatrician specializing in the care of children affected by genetic disorders [44,45,46,47]. The main indications for genetic testing for prenatal and postnatal diagnosis are summarized in Table 1.

Before the description of the principal genetic tests, some ethical considerations are mandatory.

First of all, it is of utmost importance to carefully inform the patient about the genetic test he/she is going to have. In the particular situation of children, consent has to be given by parents or legal guardians; however, if the child is old enough, and in particular, in the case of an adolescent, he/she should be involved as much as possible in the decision [48]. The patient should be aware of the possibility of unexpected secondary findings [49], particularly if whole exome sequencing (WES) or whole genome sequencing (WGS) is performed [50,51]. According to ACMG, genetic variants are divided into five classes, including pathogenic, likely pathogenic, variant of uncertain significance (VUS), likely benign, and benign [52]. VUSs are difficult to interpret, and their findings can cause uncertainty in the patient outcome.

Another consideration to take into account is that in particular, in countries with private health insurance systems, genetic test results could have repercussions on the possibility of obtaining health insurance, or even obtaining a job. To prevent this form of discrimination, in 2008, a law called the “Genetic Information Nondiscrimination Act” (GINA) was approved. According to this act, health insurers and employers are interdicted from using genetic information to make decisions about an individual’s eligibility for health insurance or a job. Nevertheless, some groups of people are not protected by this law, for example, people working for employers with less than 15 employees, people with military insurance, and people applying for long-term and life insurance [53].

The most common genetic tests and their indications, applications, and limitations are summarized in Table 2.

### 4.1. Karyotyping

Since the 1950s, karyotyping has been the first genetic test performed in patients suspected of a genetic condition. It allows us to determine the number and structure of chromosomes, detecting large structural changes, such as aneuploidies, translocations, isochromosomes, and ring chromosomes, as well as balanced changes (exchange of DNA among chromosomes through translocations, insertions, and inversions without any loss of genetic material). The main limitation is the resolution; karyotyping cannot detect microdeletions (loss of <5 Mb of genetic material). Also, it cannot detect uniparental disomy. Karyotyping is usually performed on blood cells; however, if mosaicism is suspected, performing the analysis on different tissues is indicated, usually fibroblasts [54,55,56].

### 4.2. Fluorescence In Situ Hybridization

Thanks to the introduction of fluorescence in situ hybridization (FISH), the detection of chromosomal imbalances too small to be detected by the microscope became possible. These include both microdeletions and microduplications, with the latter being harder to identify with FISH than the former. These chromosomal alterations are also known as copy number variants (CNVs), and since the 1980s, they have been more and more recognized as a cause of intellectual disability in syndromic patients [57,58].

FISH uses fluorescent probes that bind to nucleic acid sequences with a high degree of sequence complementarity, detecting deletions or duplications too small to be recognized through karyotyping. The resolution is dependent on the chosen probe (usually 50 Kb–1 Mb). The main limitation of FISH is that it is a target method; therefore, it can be helpful only if the clinician suspects a specific disease that will be investigated with targeted probes [56].

In recent years, the development of new technologies, known as chromosome microarrays, has supplanted FISH because they made it possible to interrogate the entire genome instead of a single locus [59].

### 4.3. Chromosomal Microarray

Chromosomal microarray (CMA) is used to detect chromosomal imbalances with a significantly higher resolution than routine cytogenetic analysis (karyotyping and FISH). It is recommended as a first-tier diagnostic test for patients with intellectual disabilities, neurodevelopmental delays autism associated with syndromic features, multiple congenital anomalies, and dysmorphic features [60,61,62,63,64,65].

There are two CMA techniques used to detect chromosomal imbalances, including array comparative genomic hybridization (array-CGH) and single-nucleotide polymorphism array (SNP array).

Array-CGH compares the DNA of the patient with DNA derived from normal controls. SNP array uses DNA probes derived from genomic regions characterized by differences between individuals at a single base pair site. Unlike array-CGH, SNP array can detect consanguinity (loss of heterozygosity (LOH)), triploidy, uniparental disomy (UPD), genetic identity by descent (IBD), and different cell lines (for example, cells from a twin or the mother) [66,67,68].

### 4.4. Multiplex Ligation-Dependent Probe Amplification

Multiplex ligation-dependent probe amplification (MLPA) allows us to determine the copy number of up to 60 genomic DNA sequences in a single multiplex PCR-based reaction [69]. MLPA is used for the detection of aneuploidies of chromosomes 13, 18, 21, X, and Y, (micro)deletion and (micro)duplication syndromes (in particular, Duchenne and Becker muscular dystrophy, spinal muscular atrophy, and SHOX deficiency), and the characterization of marker chromosomes [70,71].

A variant of MLPA allows for the identification of epigenetic mutations caused by the alteration of the methylation status of genes and promoter regions. Prader–Willi syndrome and Angelman syndrome are the most common genetic diseases caused by imprinting disorders, and MLPA is often used to achieve their molecular diagnosis [72]. MLPA is more rapid and less expensive and has a higher throughput than karyotyping and FISH. However, MLPA cannot detect low-grade mosaicism, female triploidies and balanced chromosomal abnormalities, such as inversions and translocations [69].

### 4.5. Next-Generation Genome Sequencing

Next-generation genome sequencing (NGS) allows for the simultaneous analysis of a great number of genes, leading to the sequencing of the whole genome in a few days. Depending on the clinical picture, it is possible to choose sequencing for a panel of genes or the whole exome or genome. Gene panels include hundreds to thousands of genes and are useful for diagnosing diseases characterized by genetic and phenotypic heterogeneity. They are available for conditions that can be caused by several known genetic mutations, such as neurological disorders, intellectual disability, early hearing loss, cardiomyopathies, skeletal dysplasia, short stature, RASopathies, and inborn errors of metabolism [73,74,75]. Because some genomic regions are difficult to sequence (for example sequences with high or low guanine-cytosine content, repetitive sequences, duplicated sequences, and pseudogenes), complementary genetic testing performed with different techniques can be required [76,77].

For patients presenting with an overlap of phenotypes that cannot be attributed to known genetic mutations, or in case of previous negative genetic testing, a wider NGS technique can be used. Whole exome sequencing (WES) analyzes the coding part of the genome, in which approximately 85% of disease-causing variants have been identified. In total, it represents 1–1.5% of the whole genome.

The diagnostic rate ranges from less than 20% to 60%, depending on the report, and is higher in cohorts of patients with a high rate of consanguinity and if trio-WES is performed (parents and affected child genome analyzed simultaneously) [78,79,80].

When multiple patients with similar phenotypes show the same genetic alteration, novel candidate genes are identified, stepping further into the knowledge of the human genome. If, initially, WES was not advised as the first-tier genetic test, decreasing costs and the increased availability of the technology in multiple laboratories are making NGS and, in particular, WES more and more used at the start of the diagnostic pathway [81,82]. However, WES cannot sequence some genomic regions (for example with repeated architecture) and noncoding variants (NCV); therefore, a negative result should not discourage the clinician from looking further into a genetic cause. Whole genome sequencing (WGS) could be used in case of a negative WES result or as an alternative to WES [83]. WGS analyzes both the coding and non-coding genome, providing the possibility of also diagnosing disorders caused by the alteration of non-coding DNA. In fact, it is known that non-coding DNA plays an important role in gene regulation and protein folding [84,85].

WGS has better performance than WES in identifying single-nucleotide variants and small insertions/deletions and a lower rate of false-positive variants, and it is able to detect more potential pathogenic variants than WES [86,87,88].

Eventually, it is necessary to underline that for some patients, NGS technologies will not be diagnostic. In these cases, it is essential to reassess the clinical picture, consider the possibility of additional genetic testing (for example SNP array), and reconsider the results in the future because genetic databases are constantly updated with new variants recognized as pathogenic [89,90].

## 5. Discussion

**Figure 2:**
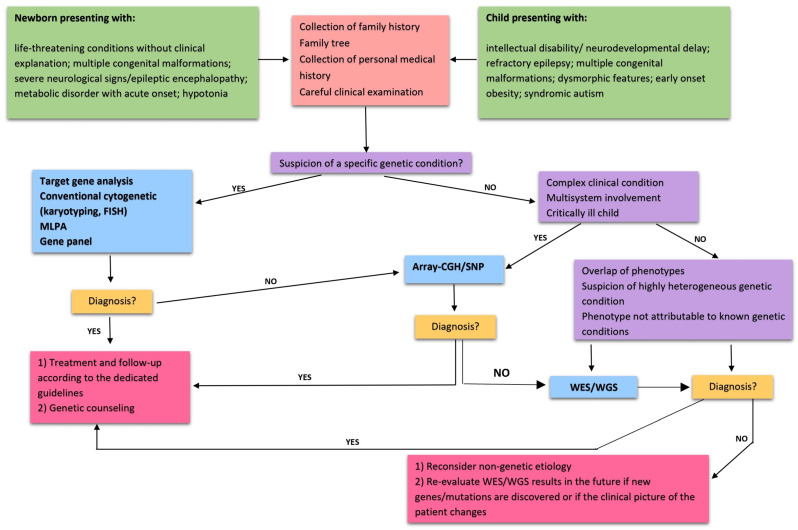
The approach to treating a child with suspicion of congenital syndrome: diagnostic algorithm.

In the paragraphs above, we aimed to offer an overview of the clinical approach to treating a child bearing features that are suggestive of a genetic syndrome. We focused on the collection of the family and medical history and the clinical features that should be noticed during the physical examination, describing in detail the systematic analysis of facial features. In fact, several genetic syndromes are characterized by a typical facial appearance, and the recognition of specific dysmorphic traits can help achieve the correct diagnosis. In this review, we also offered an overview of the most commonly used genetic tests, and we suggested some tools that are probably not known by most general pediatricians and pediatricians in training. In Figure 2, a diagnostic algorithm is offered. Clearly, the diagnosis and follow-up of specific genetic conditions and syndromes should be ordered by a geneticist or a pediatrician with expertise in the field to offer the best care possible to the child. Nevertheless, it is important for a general pediatrician to have at least some knowledge on this topic and to be able to recognize and describe red flags because early referral to the specialist is of utmost importance. Of course, it is not the intention of this manuscript to provide a comprehensive dissertation of every single aspect connected to this topic, for which the reader can refer to specific textbooks, databases, and dedicated resources (some of which have been suggested above).

## 6. Conclusions

The approach to treating a child with dysmorphic features can be challenging for pediatricians with no or little experience in rare diseases. Through the collection of the family and medical history and the physical examination, the suspicion of a genetic syndrome should be raised, and the child should be referred to a geneticist or a pediatrician specializing in the care of children affected by rare diseases. A prompt diagnosis is essential to guarantee optimal care to the patients and their families, start the screening for comorbidities, and prevent future complications. Several tools can support clinicians and be used as educational and teaching support. The appropriate genetic test must be chosen to achieve the diagnosis. Pediatricians are not required to know these technologies in detail; however, basic knowledge can be helpful in understanding the reports of genetic tests and their limitations.

## Figures and Tables

**Table 1:** Main indications for genetic testing for prenatal and postnatal diagnosis in children.

Prenatal Diagnosis	Postnatal Diagnosis
Abnormal fetal ultrasound	Abnormal clinical phenotype
Maternal serum screening or non-invasive prenatal testing (NIPT) indicating an increased risk of a fetus carrying a chromosomal abnormality	Multiple congenital abnormalities
	Intellectual disability
Parental chromosome rearrangement, mosaicism, or previous aneuploidy	Epilepsy
	Autism
Previous livebirth/stillbirth with a chromosome abnormality	Early onset obesity
Familial monogenic disorder	Early onset hearing loss
	Early onset visual problem
	Clinically significant abnormal growth (short stature, excessive growth, microcephaly, macrocephaly)
	Ambiguous genitalia
	Family history of chromosome rearrangements in a symptomatic child

**Table 2:** Main genetic tests and their indications.

Method	Main Indications in Children	Point of Strength	Limitations
Karyotyping	- Suspicion of chromosomal syndrome - Rule out structural variant after microarray findings - Aneuploidy	- Detection of large structural changes (aneuploidies, translocations, isochromosomes, rings, CNVs > 5–10 Mb) and balanced changes (translocations, insertions, rings)	- Cannot detect small rearrangements below the resolution, nucleotide variants, UPD, mosaicism < 10%
Fluorescence in situ hybridization (FISH)	- Target test in case of specific syndromes suspected (e.g., Di George syndrome) in prenatal or postnatal care - Follow-up after abnormal karyotype (e.g., SRY FISH on abnormal Y) - Prenatal aneuploidy in urgency	- Detection of aneuploidies, CNVs, translocations, inversions, insertions	- Cannot detect nucleotide variants, mosaicism < 10%, UPD - Targeted analysis: need for suspicion of a specific disease to choose the probes to be used
Chromosomal microarray (CMA): array-CGH SNP array	- Intellectual disability - Developmental delay - Syndromic autism - Multiple congenital anomalies - Dysmorphic features	- Detection of regions of homozygosity, uniparental disomy, polyploidy, mosaicism, chimerism, DNA contamination, and false paternity	- Cannot detect balanced rearrangement, mosaicism < 10% - Nucleotide variants, independent cell lines, heterochromatic markers, triploidy, and UPD can be detected using an SNP array
Multiplex Ligation-Dependent Probe Amplification (MLPA)	- Imprinting disorders (Prader–Willi syndrome, Angelman syndrome…) - Quantitative variants if described in the literature (i.e., SHOX deficiency) - Neuromuscular disorders - Aneuploidies of chromosomes 13, 18, 21, X, Y	- Detection of deletions or duplications, altered methylation of genes - More rapid and less expensive than FISH and karyotyping	- Cannot detect low-grade mosaicism, female triploidies, and copy number neutral chromosome abnormalities (inversions and translocations) - Targeted analysis: need for suspicion of a specific disease to choose the probes to be used
Gene panel	- Cardiomyopathy - Hearing loss - Intellectual disability - Short stature - Skeletal dysplasia - Inborn errors of metabolism	- Sequencing of multiple genes at the same time	- Poor identification of highly homologous regions and regions of high and low GC content - Repeated and duplicated sequences of any size cannot be sequenced with high confidence or quality
Whole Exome sequencing (WES)	- Neurodevelopmental disabilities - Multiple malformations	- Sequencing of multiple genes at the same time - Discovery of new genetic conditions	- Poor identification of sequences with extreme guanine/cytosine content or repeated architecture (e.g., Fragile X syndrome or Huntington’s disease) - Nonidentifications of genetic alteration in the non-coding portion of the genome - High costs, not reimbursed in many countries
Whole Genome sequencing (WGS)	- Multisystem involvement - Progressive clinical course - Phenotype not attributable to known genetic conditions - Differential diagnosis including two or more conditions that would be evaluated in separate panels	- Sequencing of multiple genes at the same time - Discovery of new genetic conditions - Coverage of coding and non-coding genomic regions - WGS is better than WES in identifying single-nucleotide variants and small insertions/deletions.	- Costs - Currently not widely available
